# The genome sequence of a tachinid fly, *Gymnocheta viridis *(Fallén, 1810)

**DOI:** 10.12688/wellcomeopenres.23435.1

**Published:** 2024-12-03

**Authors:** Maxwell V.L. Barclay, Steven Falk, Olga Sivell, Natural History Museum Genome Acquisition Lab, University of Oxford and Wytham Woods Genome Acquisition Lab, Darwin Tree of Life Barcoding collective, Wellcome Sanger Institute Tree of Life Management, Samples and Laboratory team, Wellcome Sanger Institute Scientific Operations: Sequencing Operations, Wellcome Sanger Institute Tree of Life Core Informatics team, Tree of Life Core Informatics collective, Darwin Tree of Life Consortium

**Affiliations:** 1 National History Museum, London, England, UK; 2 Independent researcher, Kenilworth, England, UK

**Keywords:** Gymnocheta viridis, tachinid fly, genome sequence, chromosomal, Diptera

## Abstract

We present a genome assembly from an individual male specimen of *Gymnocheta viridis* (tachinid fly; Arthropoda; Insecta; Diptera; Tachinidae). The genome sequence has a total length of 600.30 megabases. Most of the assembly (98.1%) is scaffolded into 6 chromosomal pseudomolecules, including the X sex chromosome. The mitochondrial genome has also been assembled and is 19.34 kilobases in length. Gene annotation of this assembly on Ensembl identified 12,716 protein-coding genes.

## Species taxonomy

Eukaryota; Opisthokonta; Metazoa; Eumetazoa; Bilateria; Protostomia; Ecdysozoa; Panarthropoda; Arthropoda; Mandibulata; Pancrustacea; Hexapoda; Insecta; Dicondylia; Pterygota; Neoptera; Endopterygota; Diptera; Brachycera; Muscomorpha; Eremoneura; Cyclorrhapha; Schizophora; Calyptratae; Oestroidea; Tachinidae; Tachininae; Ernestiini; *Gymnocheta*; *Gymnocheta viridis* (Fallén, 1810) (NCBI:txid631317)

## Background

*Gymnocheta viridis* is a large, bristly fly from the family Tachinidae, commonly called parasitic flies or bristle flies. *Gymnocheta* is one of two European genera with metallic green species, the other being *Chrysosomopsis* Townsend, 1916 with one rare species in Britain, *C. aurata* (Fallén, 1810) ( Belshaw, 1993; Mitchell & Raper, 2020). These species can be separated by the colour of palps: yellow in *C. aurata* and black in *G. viridis*; and the number of postsutural dorsocentrals: three in *G. viridis* and four in *C. aurata* ( Belshaw, 1993; Pohjoismäki & Bergström, 2021).

Until recently *G. viridis* was considered the only species from the genus *Gymnocheta* Robineau-Desvoidy, 1830 to occur in Britain ( Belshaw, 1993). However, the discovery of *G. lucida* Zimin, 1958 and *G. zhelochovtsevi* Zimin, 1958 from Finland and Sweden (previously only known from Russian Far East and Japan), raised questions about presence of these species in Britain. In 2022 *G. magna* Zimin, 1958 was discovered in Scotland ( Falk, 2023; Raper, 2022) and *G. lucida* Zimin, 1958, previously overlooked, was added to British list a year later ( Horsfield *et al.*, 2023).

These three species are very similar in appearance, with only minute morphological details separating them. The key to males of all British species, including detailed photographs of male genitalia, was provided by Horsfield *et al.* (2023). A key to seven (out of eight described) Palaearctic species of *Gymnocheta,* as well as detailed descriptions and illustrations of the four Nordic species mentioned above, was provided by Pohjoismäki & Bergström (2021). Pohjoismäki *et al.* (2016) published the COI barcodes of the four species from Finland (available on GenBank). It is worth noting that *G. viridis* and Finnish *G. zhelochovtsevi* are indistinguishable using CO1 barcodes ( Pohjoismäki *et al.*, 2016). More extensive DNA analysis should further our understanding of those two, morphologically distinct species.

There is a further issue with identity of *G. viridis* as the holotype was shown to fit the description of *G. magna* instead *.* A request was issued to the International Commission on Zoological Nomenclature for the replacement of the current holotype of *G. viridis* with a neotype and preservation of the commonly used species concept ( Pohjoismäki & Bergström, 2021). The identification of the specimen that was used for the genome sequence presented here follows Pohjoismäki & Bergström (2021).

*Gymnocheta viridis* is a widely distributed Palaearctic species. In Britain it is common and widespread, in contrast to *G. magna* and *G. lucida* which have been recorded only from Scotland. It is a spring-early summer flying species, recorded from March to June, occasionally July ( NBN Atlas Partnership, 2024). It occurs in woodland, forest margins, meadows and also in gardens. Males often bask on trees while females fly low among grasses, in search of a host. The larvae are parasitoids of Lepidoptera stem borers in grasses or sedges: *Mesapamea secalis* (Linnaeus, 1758), *Photedes minima* Haworth, 1809, *Denticucullus pygmina* Haworth, 1809 *and Amphipoea ussuriensis* Petersen, 1914 ( Belshaw, 1993; Tschorsnig, 2017). The adults feed on flowers of cow parsnip ( *Anthriscus sylvestris*) and other umbellifers (Apiacae) ( Pohjoismäki & Bergström, 2021).

The genome of *Gymnocheta viridis* was sequenced as part of the Darwin Tree of Life Project, a collaborative effort to sequence all named eukaryotic species in the Atlantic Archipelago of Britain and Ireland. Here we present a chromosomally complete genome sequence for *Gymnocheta viridis*, based on one specimen (NHMUK014380546, SAMEA9359420) from London, England, UK.

## Genome sequence report

**Figure 1.**
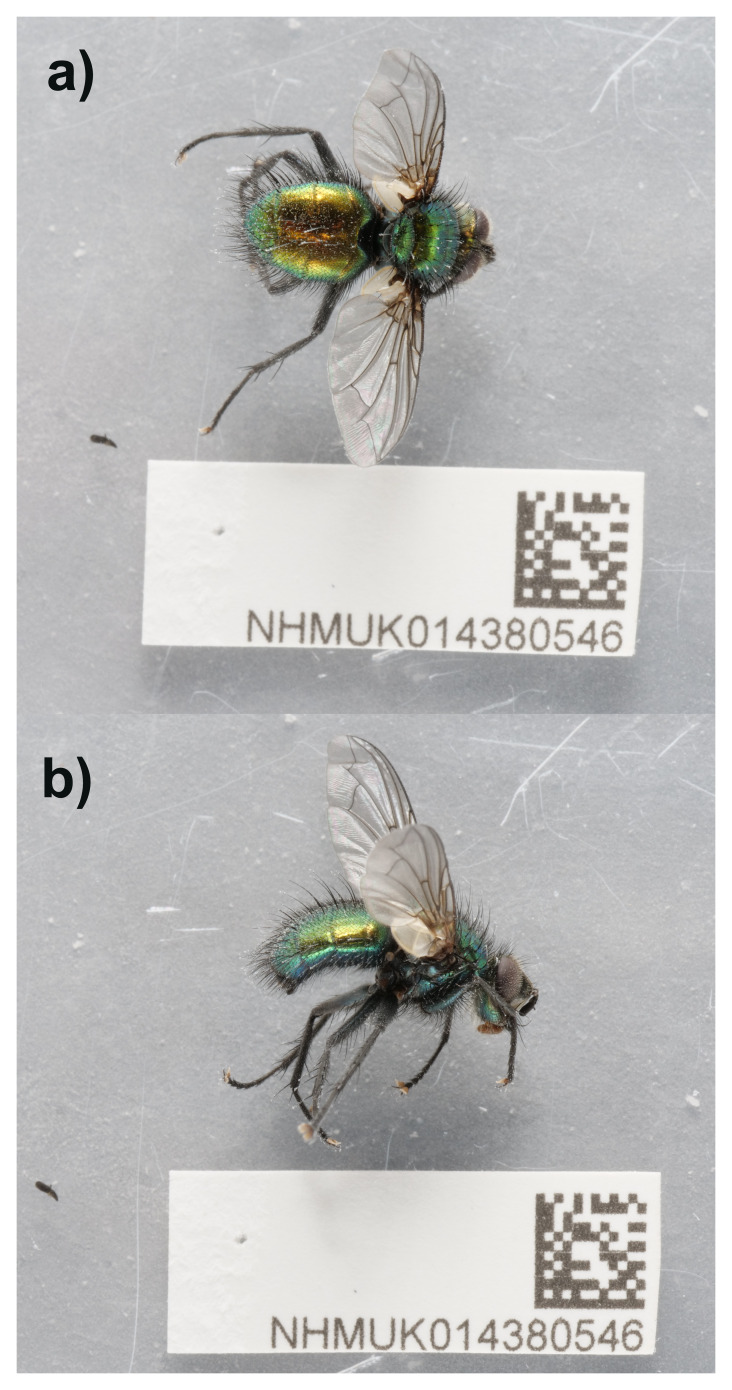
Photographs of the *Gymnocheta viridis* (idGymViri1) specimen used for genome sequencing.

The genome of *Gymnocheta viridis* ( Figure 1) was sequenced using Pacific Biosciences single-molecule HiFi long reads, generating a total of 26.80 Gb (gigabases) from 2.64 million reads, providing an estimated 47-fold coverage. Primary assembly contigs were scaffolded with chromosome conformation Hi-C data, which produced 132.29 Gb from 876.10 million reads. Specimen and sequencing details are summarised in Table 1.

**Table 1.** Specimen and sequencing data for *Gymnocheta viridis*.

Project information			
**Study title**	Gymnocheta viridis		
**Umbrella BioProject**	PRJEB61907		
**Species**	*Gymnocheta viridis*		
**BioSample**	SAMEA9359420		
**NCBI taxonomy ID**	631317		
Specimen information			
**Technology**	**ToLID**	**BioSample accession**	**Organism part**
**PacBio long read sequencing**	idGymViri1	SAMEA9359485	abdomen
**Hi-C sequencing**	idGymViri1	SAMEA9359484	head
**RNA sequencing**	idGymViri3	SAMEA10201058	abdomen
**Sequencing information**			
**Platform**	**Run accession**	**Read count**	**Base count (Gb)**
**Hi-C Illumina NovaSeq 6000**	ERR11439639	8.76e+08	132.29
**PacBio Sequel IIe**	ERR11435983	2.30e+06	23.58
**PacBio Sequel IIe**	ERR11435984	2.41e+05	2.07
**PacBio Sequel IIe**	ERR11435985	9.86e+04	1.15
**RNA Illumina NovaSeq 6000**	ERR11439640	8.57e+07	12.93

Assembly errors were corrected by manual curation, including 126 missing joins or mis-joins and two haplotypic duplications. This reduced the scaffold number by 26.81%, and increased the scaffold N50 by 100.91%. The final assembly has a total length of 600.30 Mb in 231 sequence scaffolds, with 453 gaps, and a scaffold N50 of 127.6 Mb ( Table 2).

**Table 2.** Genome assembly data for *Gymnocheta viridis*, idGymViri1.1.

Genome assembly		
Assembly name	idGymViri1.1	
Assembly accession	GCA_956483585.1	
*Accession of alternate haplotype*	*GCA_956483595.1*	
Span (Mb)	600.30	
Number of contigs	685	
Number of scaffolds	231	
Longest scaffold (Mb)	148.02	
Assembly metrics *		*Benchmark*
Contig N50 length (Mb)	2.9	*≥ 1 Mb*
Scaffold N50 length (Mb)	127.6	*= chromosome N50*
Consensus quality (QV)	59.0	*≥ 40*
*k*-mer completeness	100.0%	*≥ 95%*
BUSCO **	C:98.7%[S:98.2%,D:0.5%], F:0.4%,M:0.9%,n:3,285	*S > 90%, D < 5%*
Percentage of assembly mapped to chromosomes	98.1%	*≥ 90%*
Sex chromosomes	X	*localised homologous pairs*
Organelles	Mitochondrial genome: 19.34 kb	*complete single alleles*
Genome annotation of assembly GCA_956483585.1 at Ensembl		
Number of protein-coding genes	12,716	
Number of non-coding genes	2,342	
Number of gene transcripts	22,138	

* Assembly metric benchmarks are adapted from Rhie *et al.* (2021) and the Earth BioGenome Project Report on Assembly Standards September 2024. ** BUSCO scores based on the diptera_odb10 BUSCO set using version 5.3.2. C = complete [S = single copy, D = duplicated], F = fragmented, M = missing, n = number of orthologues in comparison. A full set of BUSCO scores is available at https://blobtoolkit.genomehubs.org/view/idGymViri1_1/dataset/idGymViri1_1/busco.

**Figure 2.**
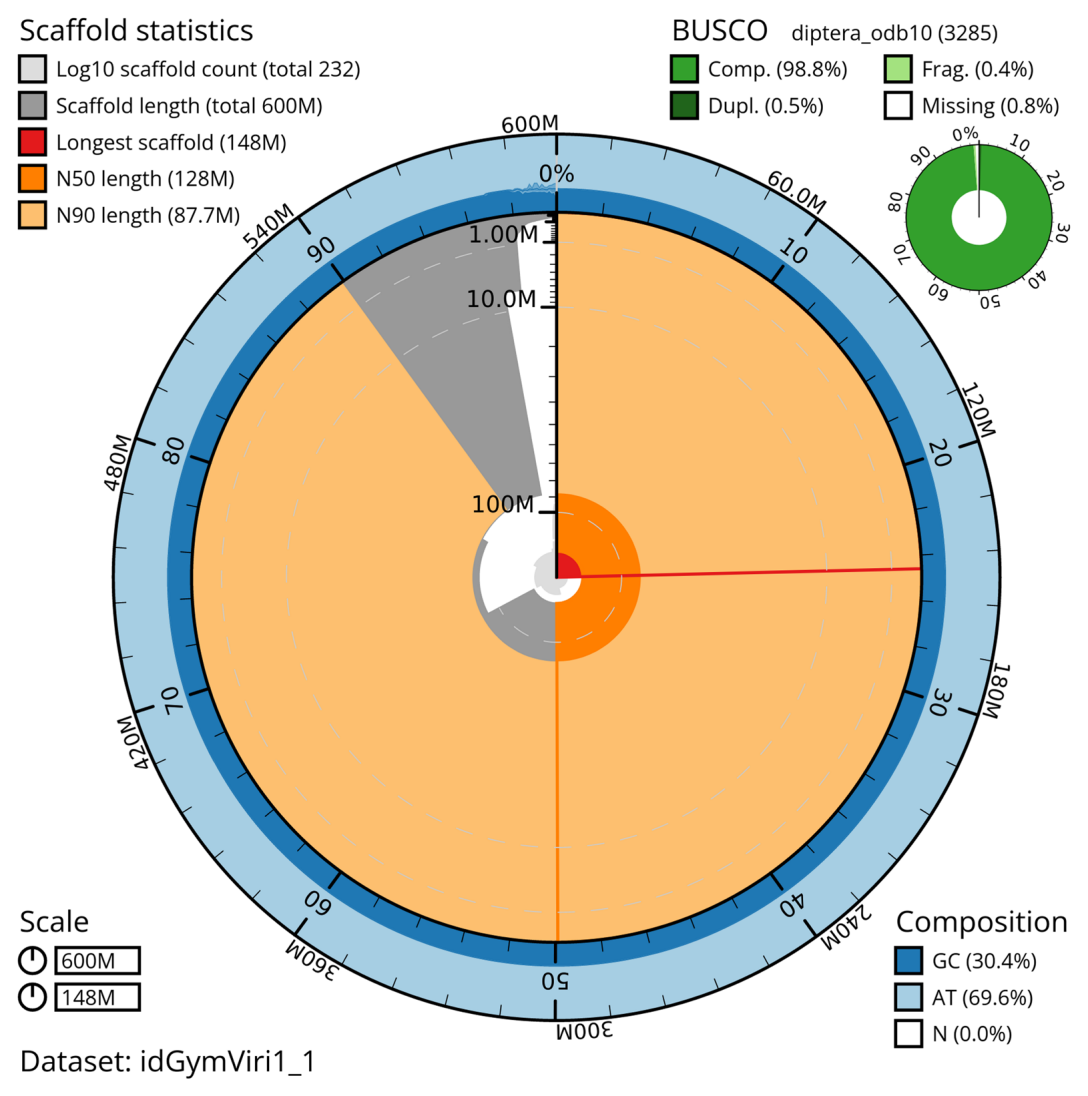
Genome assembly of *Gymnocheta viridis*, idGymViri1.1: metrics. The BlobToolKit snail plot provides an overview of assembly metrics and BUSCO gene completeness. The circumference represents the length of the whole genome sequence, and the main plot is divided into 1,000 equal-sized bins around the circumference. The outermost blue tracks display the distribution of GC, AT, and N percentages across the bins. Scaffolds are arranged clockwise from longest to shortest and are depicted in dark grey. The longest scaffold is indicated by the red arc, and the deeper orange and pale orange arcs represent the N50 and N90 lengths. A light grey spiral at the centre shows the cumulative scaffold count on a logarithmic scale. A summary of complete, fragmented, duplicated, and missing BUSCO genes in the diptera_odb10 set is presented at the top right. An interactive version of this figure is available at https://blobtoolkit.genomehubs.org/view/idGymViri1_1/dataset/idGymViri1_1/snail.

**Figure 3.**
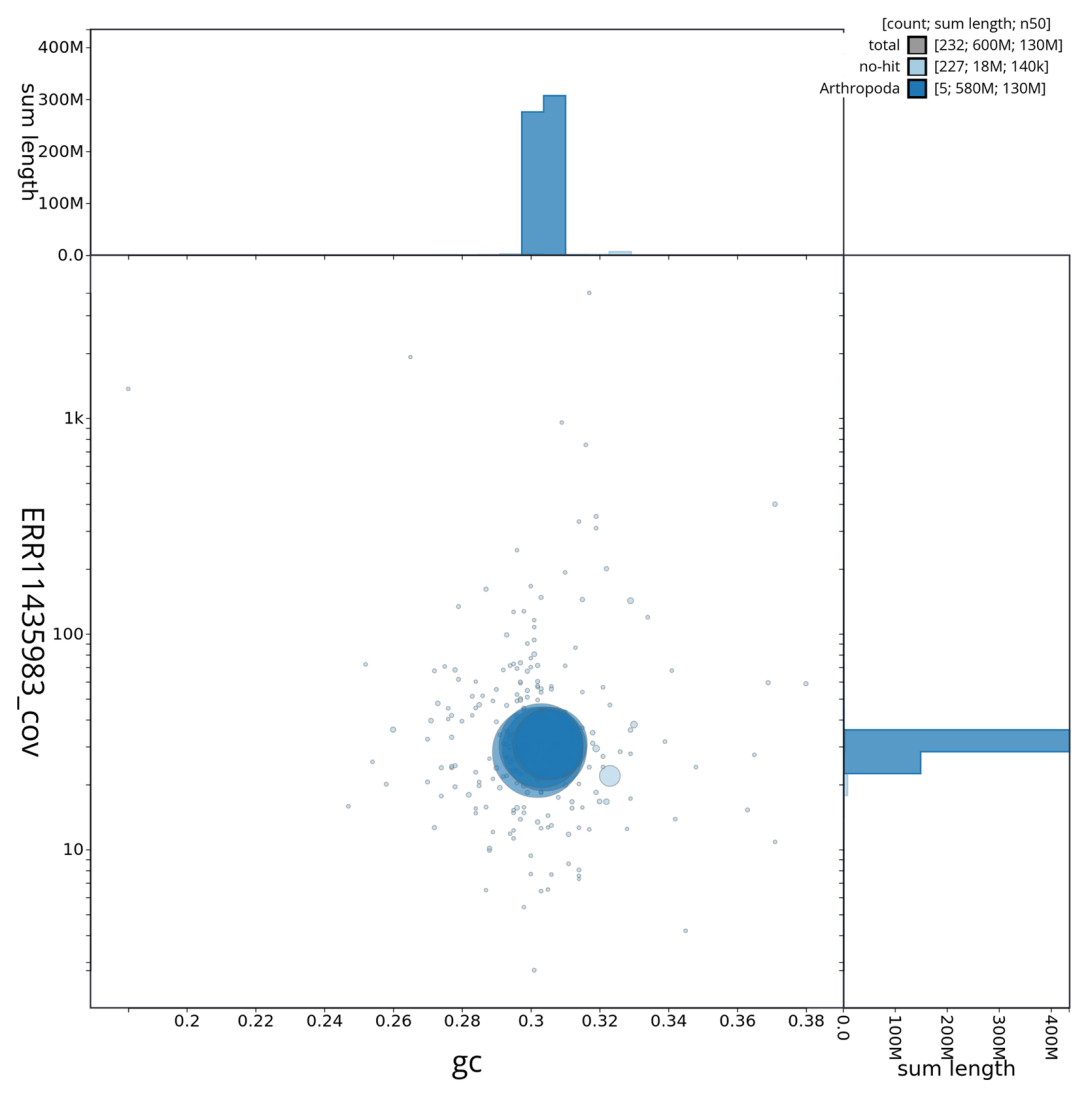
Genome assembly of *Gymnocheta viridis*, idGymViri1.1: BlobToolKit GC-coverage plot. BlobToolKit GC-coverage plot showing sequence coverage (vertical axis) and GC content (horizontal axis). The circles represent scaffolds, with the size proportional to scaffold length and the colour representing phylum membership. The histograms along the axes display the total length of sequences distributed across different levels of coverage and GC content. An interactive version of this figure is available at https://blobtoolkit.genomehubs.org/view/idGymViri1_1/dataset/idGymViri1_1/blob.

**Figure 4.**
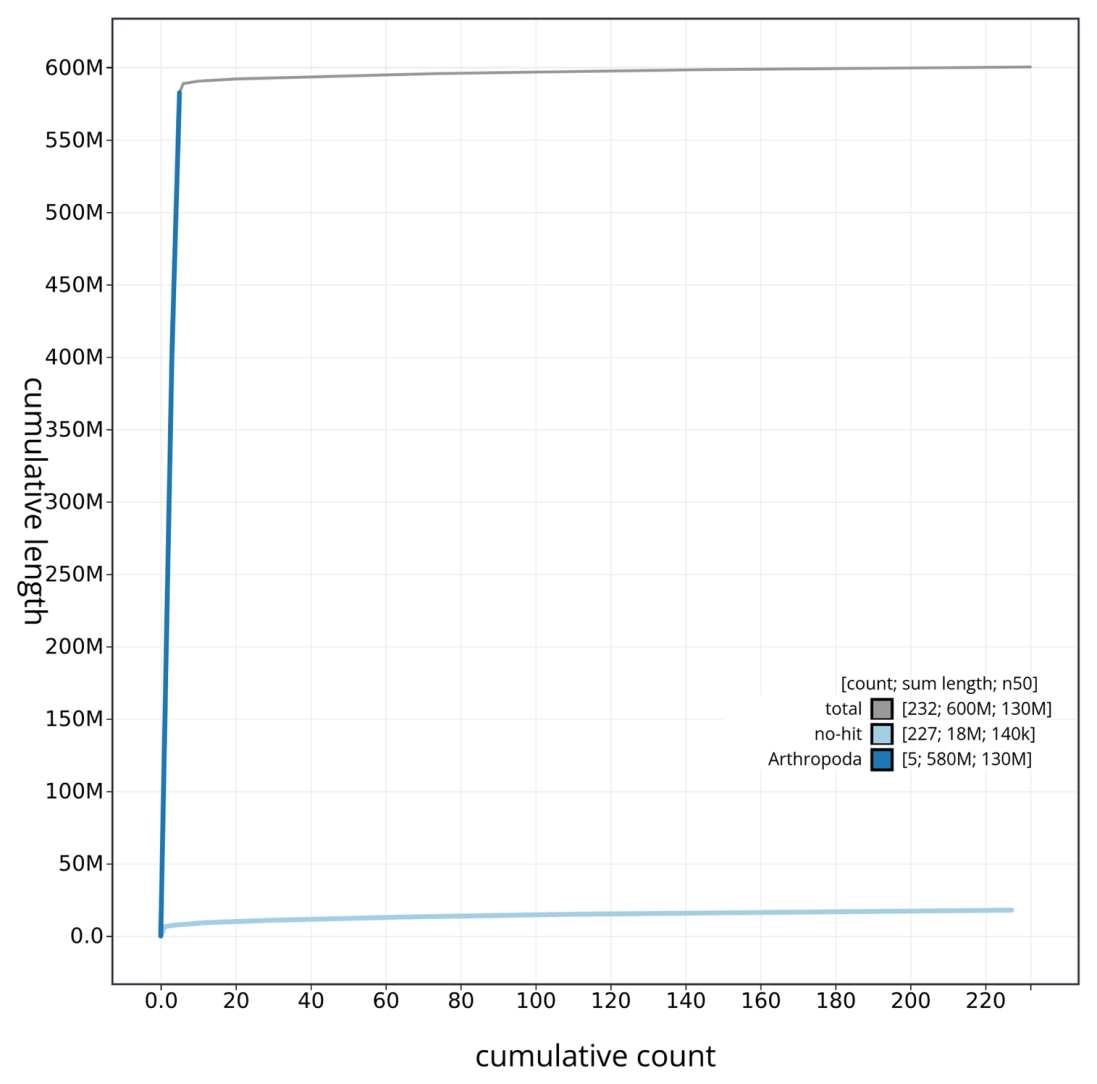
Genome assembly of *Gymnocheta viridis* idGymViri1.1: BlobToolKit cumulative sequence plot. The grey line shows cumulative length for all sequences. Coloured lines show cumulative lengths of sequences assigned to each phylum using the buscogenes taxrule. An interactive version of this figure is available at https://blobtoolkit.genomehubs.org/view/idGymViri1_1/dataset/idGymViri1_1/cumulative.

The snail plot in Figure 2 provides a summary of the assembly statistics, indicating the distribution of scaffold lengths and other assembly metrics. Figure 3 shows the distribution of scaffolds by GC proportion and coverage. Figure 4 presents a cumulative assembly plot, with separate curves representing different scaffold subsets assigned to various phyla, illustrating the completeness of the assembly.

**Figure 5.**
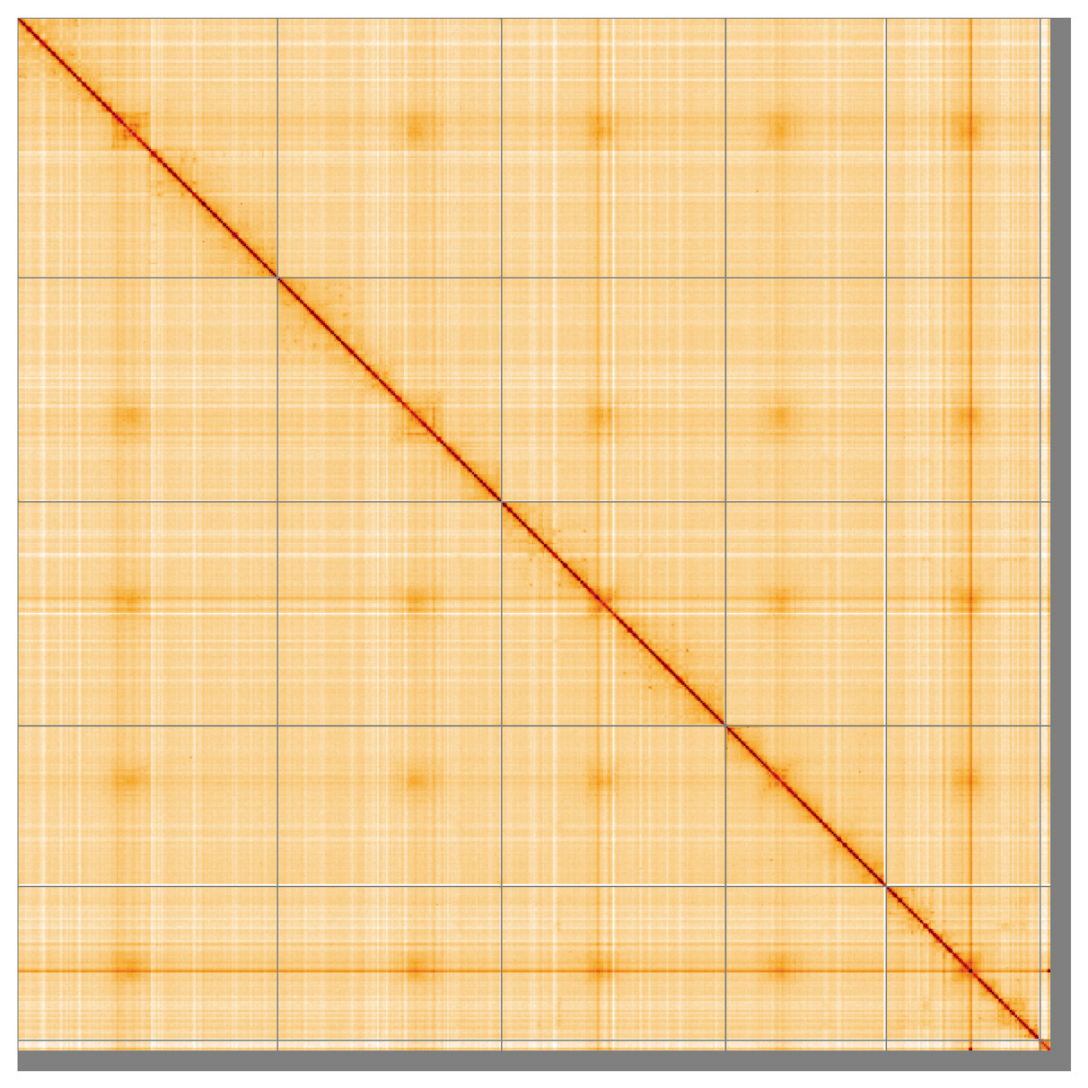
Genome assembly of *Gymnocheta viridis* idGymViri1.1: Hi-C contact map of the idGymViri1.1 assembly, visualised using HiGlass. Chromosomes are shown in order of size from left to right and top to bottom. An interactive version of this figure may be viewed at https://genome-note-higlass.tol.sanger.ac.uk/l/?d=XWLrGnOaSYW1tNme6lTGVw.

Most of the assembly sequence (98.1%) was assigned to 6 chromosomal-level scaffolds, representing 5 autosomes and the X sex chromosome. These chromosome-level scaffolds, confirmed by the Hi-C data, are named in order of size ( Figure 5; Table 3). During manual curation, chromosome X was assigned based on read coverage statistics. The species appears to be XO. The following regions of the assembly are of uncertain order and orientation: Chromosome 1: 60.5–69.5 Mb, Chromosome 5: 43.4–52.6 Mbp.

**Table 3.** Chromosomal pseudomolecules in the genome assembly of *Gymnocheta viridis*, idGymViri1.

INSDC accession	Name	Length (Mb)	GC%
OY101440.1	1	148.02	30.0
OY101441.1	2	127.73	30.5
OY101442.1	3	127.57	30.5
OY101443.1	4	91.49	30.5
OY101444.1	5	87.67	30.5
OY101445.1	X	6.34	32.5
OY101446.1	MT	0.02	18.5

While not fully phased, the assembly deposited is of one haplotype. Contigs corresponding to the second haplotype have also been deposited. The mitochondrial genome was also assembled and can be found as a contig within the multifasta file of the genome submission, and as a separate fasta file with accession OY101446.1.

The final assembly has a Quality Value (QV) of 59.0 and *k*-mer completeness of 100.0%. BUSCO (v5.4.3) analysis using the diptera_odb10 reference set ( *n* = 3,285) indicated a completeness score of 98.7% (single = 98.2%, duplicated = 0.5%). Other quality metrics are given in Table 2.

## Genome annotation report

The *Gymnocheta viridis* genome assembly (GCA_956483585.1) was annotated at the European Bioinformatics Institute (EBI) on Ensembl Rapid Release. The resulting annotation includes 22,138 transcribed mRNAs from 12,716 protein-coding and 2,342 non-coding genes ( Table 2; https://rapid.ensembl.org/Gymnocheta_viridis_GCA_956483585.1/Info/Index). The average transcript length is 16,522.03. There are 1.47 coding transcripts per gene and 4.96 exons per transcript.

## Methods

### Sample acquisition and DNA barcoding

An adult male specimen of *Gymnocheta viridis* (specimen ID NHMUK014380546, ToLID idGymViri1) was collected from Maltings Place, Fulham, London, United Kingdom (latitude 51.48, longitude –0.19) on 2021-04-13, using an aerial net. The specimen was collected and identified by Maxwell Barclay (Natural History Museum) and preserved by dry freezing at –80 °C.

The specimen used for RNA sequencing (specimen ID Ox001319, ToLID idGymViri3) was an adult specimen collected from Wytham Woods, Oxfordshire, United Kingdom (latitude 51.76, longitude –1.34) on 2021-04-23 by netting. The specimen was collected and identified by Steven Falk (independent researcher) and preserved on dry ice.

The initial identification was verified by an additional DNA barcoding process according to the framework developed by Twyford *et al.* (2024). A small sample was dissected from the specimens and stored in ethanol, while the remaining parts were shipped on dry ice to the Wellcome Sanger Institute (WSI) ( Pereira *et al.*, 2022). The tissue was lysed, the COI marker region was amplified by PCR, and amplicons were sequenced and compared to the BOLD database, confirming the species identification ( Crowley *et al.*, 2023). Following whole genome sequence generation, the relevant DNA barcode region was also used alongside the initial barcoding data for sample tracking at the WSI ( Twyford *et al.*, 2024). The standard operating procedures for Darwin Tree of Life barcoding have been deposited on protocols.io ( Beasley *et al.*, 2023).

### Nucleic acid extraction

The workflow for high molecular weight (HMW) DNA extraction at the WSI Tree of Life Core Laboratory includes a sequence of procedures: sample preparation and homogenisation, DNA extraction, fragmentation and purification. Detailed protocols are available on protocols.io ( Denton *et al.*, 2023b).

The idGymViri1 sample was prepared for DNA extraction by weighing and dissecting it on dry ice ( Jay *et al.*, 2023). Tissue from the abdomen was homogenised using a PowerMasher II tissue disruptor ( Denton *et al.*, 2023a).

HMW DNA was extracted using the Automated MagAttract v1 protocol ( Sheerin *et al.*, 2023). DNA was sheared into an average fragment size of 12–20 kb in a Megaruptor 3 system ( Todorovic *et al.*, 2023). Sheared DNA was purified by solid-phase reversible immobilisation, using AMPure PB beads to eliminate shorter fragments and concentrate the DNA ( Strickland *et al.*, 2023). The concentration of the sheared and purified DNA was assessed using a Nanodrop spectrophotometer and a Qubit Fluorometer using the Qubit dsDNA High Sensitivity Assay kit. The fragment size distribution was evaluated by running the sample on the FemtoPulse system.

RNA was extracted from abdomen tissue of idGymViri3 in the Tree of Life Laboratory at the WSI using the RNA Extraction: Automated MagMax™ *mir*Vana protocol ( do Amaral *et al.*, 2023). The RNA concentration was assessed using a Nanodrop spectrophotometer and a Qubit Fluorometer using the Qubit RNA Broad-Range Assay kit. Analysis of the integrity of the RNA was done using the Agilent RNA 6000 Pico Kit and Eukaryotic Total RNA assay.

### Hi-C preparation

Tissue from the head of the idGymViri1 sample was processed at the WSI Scientific Operations core, using the Arima-HiC v2 kit. Tissue (stored at –80 °C) was fixed, and the DNA crosslinked using a TC buffer with 22% formaldehyde. After crosslinking, the tissue was homogenised using the Diagnocine Power Masher-II and BioMasher-II tubes and pestles. Following the kit manufacturer's instructions, crosslinked DNA was digested using a restriction enzyme master mix. The 5’-overhangs were then filled in and labelled with biotinylated nucleotides and proximally ligated. An overnight incubation was carried out for enzymes to digest remaining proteins and for crosslinks to reverse. A clean up was performed with SPRIselect beads prior to library preparation.

### Library preparation and sequencing

Library preparation and sequencing were performed at the WSI Scientific Operations core. Pacific Biosciences HiFi circular consensus DNA sequencing libraries were prepared using the PacBio Express Template Preparation Kit v2.0 (Pacific Biosciences, California, USA) as per the manufacturer's instructions. The kit includes the reagents required for removal of single-strand overhangs, DNA damage repair, end repair/A-tailing, adapter ligation, and nuclease treatment. Library preparation also included a library purification step using AMPure PB beads (Pacific Biosciences, California, USA) and size selection step to remove templates shorter than 3 kb using AMPure PB modified SPRI. DNA concentration was quantified using the Qubit Fluorometer v2.0 and Qubit HS Assay Kit and the final library fragment size analysis was carried out using the Agilent Femto Pulse Automated Pulsed Field CE Instrument and gDNA 165kb gDNA and 55kb BAC analysis kit. Samples were sequenced using the Sequel IIe system (Pacific Biosciences, California, USA). The concentration of the library loaded onto the Sequel IIe was in the range 40–135 pM. The SMRT link software, a PacBio web-based end-to-end workflow manager, was used to set-up and monitor the run, as well as perform primary and secondary analysis of the data upon completion.

For Hi-C library preparation, DNA was fragmented to a size of 400 to 600 bp using a Covaris E220 sonicator. The DNA was then enriched, barcoded, and amplified using the NEBNext Ultra II DNA Library Prep Kit following manufacturers’ instructions. The Hi-C sequencing was performed using paired-end sequencing with a read length of 150 bp on an Illumina NovaSeq 6000 instrument.

Poly(A) RNA-Seq libraries were constructed using the NEB Ultra II RNA Library Prep kit, following the manufacturer’s instructions. RNA sequencing was performed on the Illumina NovaSeq 6000 instrument.

### Genome assembly, curation and evaluation

***Assembly***

The HiFi reads were first assembled using Hifiasm ( Cheng *et al.*, 2021) with the --primary option. Haplotypic duplications were identified and removed using purge_dups ( Guan *et al.*, 2020). The Hi-C reads were mapped to the primary contigs using bwa-mem2 ( Vasimuddin *et al.*, 2019). The contigs were further scaffolded using the provided Hi-C data ( Rao *et al.*, 2014) in YaHS ( Zhou *et al.*, 2023) using the --break option for handling potential misassemblies. The scaffolded assemblies were evaluated using Gfastats ( Formenti *et al.*, 2022), BUSCO ( Manni *et al.*, 2021) and MERQURY.FK ( Rhie *et al.*, 2020).

The mitochondrial genome was assembled using MitoHiFi ( Uliano-Silva *et al.*, 2023), which runs MitoFinder ( Allio *et al.*, 2020) and uses these annotations to select the final mitochondrial contig and to ensure the general quality of the sequence.

***Assembly curation***

The assembly was decontaminated using the Assembly Screen for Cobionts and Contaminants (ASCC) pipeline (article in preparation). Manual curation was primarily conducted using PretextView ( Harry, 2022), with additional insights provided by JBrowse2 ( Diesh *et al.*, 2023) and HiGlass ( Kerpedjiev *et al.*, 2018). Scaffolds were visually inspected and corrected as described by Howe *et al.* (2021). Any identified contamination, missed joins, and mis-joins were corrected, and duplicate sequences were tagged and removed. The sex chromosome was identified based on read coverage statistics. The curation process is documented at https://gitlab.com/wtsi-grit/rapid-curation (article in preparation).

***Evaluation of the final assembly***

A Hi-C map for the final assembly was produced using bwa-mem2 ( Vasimuddin *et al.*, 2019) in the Cooler file format ( Abdennur & Mirny, 2020). To assess the assembly metrics, the *k*-mer completeness and QV consensus quality values were calculated in Merqury ( Rhie *et al.*, 2020). This work was done using Nextflow ( Di Tommaso *et al.*, 2017) DSL2 pipelines “sanger-tol/readmapping” ( Surana *et al.*, 2023a) and “sanger-tol/genomenote” ( Surana *et al.*, 2023b). These genome evaluation pipelines were developed using nf-core tooling ( Ewels *et al.*, 2020) and MultiQC ( Ewels *et al.*, 2016), relying on the Conda package manager, the Bioconda initiative ( Grüning *et al.*, 2018), the Biocontainers infrastructure ( da Veiga Leprevost *et al.*, 2017), as well as the Docker ( Merkel, 2014) and Singularity ( Kurtzer *et al.*, 2017) containerisation solutions. The genome was also analysed within the BlobToolKit environment ( Challis *et al.*, 2020) and BUSCO scores ( Manni *et al.*, 2021) were calculated.

Table 4 contains a list of relevant software tool versions and sources.

**Table 4.** Software tools: versions and sources.

Software tool	Version	Source
BlobToolKit	4.2.1	https://github.com/blobtoolkit/blobtoolkit
BUSCO	5.3.2	https://gitlab.com/ezlab/busco
bwa-mem2	2.2.1	https://github.com/bwa-mem2/bwa-mem2
Cooler	0.8.11	https://github.com/open2c/cooler
FastK	427104ea91c78c3b8b8b49f1a7d6bbeaa869ba1c	https://github.com/thegenemyers/FASTK
Gfastats	1.3.6	https://github.com/vgl-hub/gfastats
Hifiasm	0.16.1-r375	https://github.com/chhylp123/hifiasm
HiGlass	44086069ee7d4d3f6f3f0012569789ec138f42b84aa44357826c0b6753eb28de	https://github.com/higlass/higlass
Merqury.FK	d00d98157618f4e8d1a9190026b19b471055b22e	https://github.com/thegenemyers/MERQURY.FK
MitoHiFi	2	https://github.com/marcelauliano/MitoHiFi
PretextView	0.2	https://github.com/wtsi-hpag/PretextView
purge_dups	1.2.3	https://github.com/dfguan/purge_dups
sanger-tol/ascc	-	https://github.com/sanger-tol/ascc
sanger-tol/genomenote	v1.0	https://github.com/sanger-tol/genomenote
sanger-tol/readmapping	1.1.0	https://github.com/sanger-tol/readmapping/tree/1.1.0
YaHS	yahs-1.1.91eebc2	https://github.com/c-zhou/yahs

### Genome annotation

The Ensembl Genebuild annotation system ( Aken *et al.*, 2016) was used to generate annotation for the *Gymnocheta viridis* assembly (GCA_956483585.1) in Ensembl Rapid Release at the EBI. Annotation was created primarily through alignment of transcriptomic data to the genome, with gap filling via protein-to-genome alignments of a select set of proteins from UniProt ( UniProt Consortium, 2019).

### Wellcome Sanger Institute – Legal and Governance

The materials that have contributed to this genome note have been supplied by a Darwin Tree of Life Partner. The submission of materials by a Darwin Tree of Life Partner is subject to the **‘Darwin Tree of Life Project Sampling Code of Practice’**, which can be found in full on the Darwin Tree of Life website here. By agreeing with and signing up to the Sampling Code of Practice, the Darwin Tree of Life Partner agrees they will meet the legal and ethical requirements and standards set out within this document in respect of all samples acquired for, and supplied to, the Darwin Tree of Life Project.

Further, the Wellcome Sanger Institute employs a process whereby due diligence is carried out proportionate to the nature of the materials themselves, and the circumstances under which they have been/are to be collected and provided for use. The purpose of this is to address and mitigate any potential legal and/or ethical implications of receipt and use of the materials as part of the research project, and to ensure that in doing so we align with best practice wherever possible. The overarching areas of consideration are:

•    Ethical review of provenance and sourcing of the material

•    Legality of collection, transfer and use (national and international)

Each transfer of samples is further undertaken according to a Research Collaboration Agreement or Material Transfer Agreement entered into by the Darwin Tree of Life Partner, Genome Research Limited (operating as the Wellcome Sanger Institute), and in some circumstances other Darwin Tree of Life collaborators.

## Data Availability

European Nucleotide Archive: Gymnocheta viridis. Accession number PRJEB61907; https://identifiers.org/ena.embl/PRJEB61907. The genome sequence is released openly for reuse. The *Gymnocheta viridis* genome sequencing initiative is part of the Darwin Tree of Life (DToL) project. All raw sequence data and the assembly have been deposited in INSDC databases. Raw data and assembly accession identifiers are reported in Table 1 and Table 2. Metadata for specimens, BOLD barcode results, spectra estimates, sequencing runs, contaminants and pre-curation assembly statistics are given at https://links.tol.sanger.ac.uk/species/631317.

## Author information

Members of the Natural History Museum Genome Acquisition Lab are listed here: https://doi.org/10.5281/zenodo.12159242.

Members of the University of Oxford and Wytham Woods Genome Acquisition Lab are listed here: https://doi.org/10.5281/zenodo.12157525.

Members of the Darwin Tree of Life Barcoding collective are listed here: https://doi.org/10.5281/zenodo.12158331.

Members of the Wellcome Sanger Institute Tree of Life Management, Samples and Laboratory team are listed here: https://doi.org/10.5281/zenodo.12162482.

Members of Wellcome Sanger Institute Scientific Operations: Sequencing Operations are listed here: https://doi.org/10.5281/zenodo.12165051.

Members of the Wellcome Sanger Institute Tree of Life Core Informatics team are listed here: https://doi.org/10.5281/zenodo.12160324.

Members of the Tree of Life Core Informatics collective are listed here: https://doi.org/10.5281/zenodo.12205391.

Members of the Darwin Tree of Life Consortium are listed here: https://doi.org/10.5281/zenodo.4783558.
