# Supplementary material for: The genome sequence of a tachinid fly, Gymnocheta viridis (Fallén, 1810)
Source: Wellcome Open Res. 2024 Dec 3;9:714. [Version 1] doi: 10.12688/wellcomeopenres.23435.1 (PMC11809156; doi:10.12688/wellcomeopenres.23435.1)
Supplement: Peer review history

# Reviewer report: Reviewer response for version 1

**DOI:** 10.21956/wellcomeopenres.25845.r114400

**Published:** 2025-01-21

**Authors:** Marcus C Stensmyr

**Affiliations:** 1 Lund University, Lund, Sweden

The manuscript presents a genome assembly of the bristle fly *Gymnocheta viridis, *as part of the Darwin Tree of Life (DTOL) project. The genome assembly is 600mb, scaffolded into 6 contigs of chromosome length.  Ensembl pipeline annotation found 12,716 protein-coding genes. The genome is sequenced, assembled, and annotated using the standard DTOL pipeline, and accordingly of high quality. Bristle flies are an intriguing group of flies, and a chromosome-length assembly of a *Gymnocheta* a welcome addition to diptera research.

Are sufficient details of methods and materials provided to allow replication by others?

Yes

Is the rationale for creating the dataset(s) clearly described?

Yes

Are the datasets clearly presented in a useable and accessible format?

Yes

Are the protocols appropriate and is the work technically sound?

Yes

Reviewer Expertise:

Diptera genomics and neurogenetics

I confirm that I have read this submission and believe that I have an appropriate level of expertise to confirm that it is of an acceptable scientific standard.

# Reviewer report: Reviewer response for version 1

**DOI:** 10.21956/wellcomeopenres.25845.r116992

**Published:** 2025-01-08

**Authors:** Jaakko Pohjoismäki

**Affiliations:** 1 University of Eastern Finland, Joensuu, Finland

Maxwell Barclay, Steven Falk, and Olga Sivell, in collaboration with members and associates of the DToL consortium, have successfully sequenced, assembled, and annotated a reference-quality genome assembly for the tachinid fly *Gymnocheta viridis* (Diptera: Tachinidae). This genome assembly is another nice contribution to the growing collection of high-quality genome assemblies for Oestroid flies, a group of considerable ecological and evolutionary interest.

 The species is a nice pick for a reference genome assembly as it is widespread and relatively common throughout temperate Europe. As *G. viridis* is a member of the species-rich Ernestiini tribe, the genome assembly also serves as a representative for a diverse lineage within the Tachinidae family.

 In addition to its ecological and evolutionary significance, this genome assembly is expected to aid future comparative genomic studies. By integrating this assembly with data from other Oestroid species, researchers can investigate patterns of genome evolution, gene family expansion, divergence and evolution of parasitism across this diverse group of flies. 

 Overall, the report is well written and detailed. The genome assembly meets the EBP criteria for a reference-quality genome assembly. This could actually be pointed out and referenced in the text, to aid non-specialist readers. I have only few suggestions for improvement.

**Background**

 A minor detail: the reference for requesting the preservation of the current concept for G. viridis is Pohjoismäki & Bergström, 2023, https://doi.org/10.21805/bzn.v80.a014

 Otherwise, the background provides a very nice introduction to the species and its biology.

**Genome sequence report**

 Comparison to the EBP reference genome assembly standards could be added here. It might be also worth mentioning and referencing the error rate of PacBio HiFi and its relevance to the obtained 47X genome coverage.

 Overall, based on the shown metrics, the genome assembly is of very good quality. I note that the assembly is already visible in the NCBI's genome viewer. Unfortunately most of the annotated genes do not have names (yet?), just ensemble numbers, therefore searching for known fly gene orthologs yields no hits. This seems to be often the case with non-mammalian organisms. I hope that the annotations will be completed in the future.

**Methods**

 What is an 'aerial net'? A handheld net? Does dry freezing mean that the specimen was deposited alive into -80°C freezer?

 Again, very nice work from the DToL consortium. Many thanks for covering this species.

Are sufficient details of methods and materials provided to allow replication by others?

Yes

Is the rationale for creating the dataset(s) clearly described?

Yes

Are the datasets clearly presented in a useable and accessible format?

Yes

Are the protocols appropriate and is the work technically sound?

Yes

Reviewer Expertise:

Molecular biology, biodiversity genomics, taxonomy.

I confirm that I have read this submission and believe that I have an appropriate level of expertise to confirm that it is of an acceptable scientific standard.

## References

[1] : Case 3879 – Tachina viridis Fallén, 1810 (currently Gymnocheta viridis; Insecta, Diptera, Tachinidae): proposed conservation of current usage by designation of a neotype. *The Bulletin of Zoological Nomenclature* .2023;80(1) : 10.21805/bzn.v80.a014 10.21805/bzn.v80.a014

# Reviewer report: Reviewer response for version 1

**DOI:** 10.21956/wellcomeopenres.25845.r114398

**Published:** 2024-12-12

**Authors:** Hans-Peter Fuehrer

**Affiliations:** 1 University of Veterinary Medicine, Vienna, Austria

The genome sequence of *Gymnocheta viridis* is described. One male fly was analyzed within the Darwin Tree of Life Project.

 The biology of this dipteran species is described well.

 Please describe the sampling site if possible.

 Does this tachinid fly species have no English name (because in German it has one)?

Are sufficient details of methods and materials provided to allow replication by others?

Yes

Is the rationale for creating the dataset(s) clearly described?

Yes

Are the datasets clearly presented in a useable and accessible format?

Yes

Are the protocols appropriate and is the work technically sound?

Yes

Reviewer Expertise:

Medical Entomology/Parasitology

I confirm that I have read this submission and believe that I have an appropriate level of expertise to confirm that it is of an acceptable scientific standard.
